# Robust Formation and Maintenance of Continuous Stratified Cortical Neuroepithelium by Laminin-Containing Matrix in Mouse ES Cell Culture

**DOI:** 10.1371/journal.pone.0053024

**Published:** 2012-12-31

**Authors:** Makoto Nasu, Nozomu Takata, Teruko Danjo, Hideya Sakaguchi, Taisuke Kadoshima, Sugiko Futaki, Kiyotoshi Sekiguchi, Mototsugu Eiraku, Yoshiki Sasai

**Affiliations:** 1 Organogenesis and Neurogenesis Group, Center for Developmental Biology, RIKEN, Kobe, Japan; 2 Four-Dimensional Tissue Analysis Unit, Center for Developmental Biology, RIKEN, Kobe, Japan; 3 Laboratory of Extracellular Matrix Biochemistry, Institute for Protein Research, Osaka University, Suita, Japan; University of Colorado, Boulder, United States of America

## Abstract

In the mammalian cortex, the dorsal telencephalon exhibits a characteristic stratified structure. We previously reported that three-dimensional (3D) culture of mouse ES cells (mESCs) can efficiently generate cortical neuroepithelium (NE) and layer-specific cortical neurons. However, the cortical NE generated in this mESC culture was structurally unstable and broke into small neural rosettes by culture day 7, suggesting that some factors for reinforcing the structural integrity were missing. Here we report substantial supporting effects of the extracellular matrix (ECM) protein laminin on the continuous formation of properly polarized cortical NE in floating aggregate culture of mESCs. The addition of purified laminin and entactin (a laminin-associated protein), even at low concentrations, stabilized the formation of continuous cortical NE as well as the maintenance of basement membrane and prevented rosette formation. Treatment with the neutralizing ß1-integrin antibody impaired the continuous NE formation. The stabilized cortical NE exhibited typical interkinetic nuclear migration of cortical progenitors, as seen in the embryonic cortex. The laminin-treated cortical NE maintained a continuous structure even on culture days 12 and 15, and contained ventricular, basal-progenitor, cortical-plate and Cajal-Retzius cell layers. The cortical NE in this culture was flanked by cortical hem-like tissue. Furthermore, when Shh was added, ventral telencephalic structures such as lateral ganglionic eminence–like tissue formed in the region adjacent to the cortical NE. Thus, our results indicate that laminin-entactin ECM promotes the formation of structurally stable telencephalic tissues in 3D ESC culture, and supports the morphogenetic recapitulation of cortical development.

## Introduction

The brain is the most complex organ in the vertebrate body. During early neural development, the anlage of the central nervous system (CNS) forms as the neural plate, consisting of a monolayered neuroectoderm within the dorsal ectoderm. Subsequently, the neural plate invaginates and rolls up into the neural tube, which then subdivides into the prosencephalon (forebrain), mesencephalon (midbrain), rhombencephalon (hindbrain) and spinal cord. The forebrain comprises the telencephalon and diencephalon. In addition to this anterior-posterior (AP) specification, the neural tube is patterned into the roof plate (dorsal-most), alar plate (dorsal), basal plate (ventral) and floor plate (ventral-most) along the dorsal-ventral (DV) axis. The DV pattern in the telencephalon is complex and this brain region is subdivided into the dorsal telencephalon (pallium) and ventral telencephalon (subpallium). The cerebral cortex is the largest portion of the pallium, whereas the subpallium is further regionalized into the lateral ganglionic eminance (LGE; giving rise to striatum) and the medial ganglionic eminance (MGE; giving rise to globus pallidus) [1]–[3].

In the mammalian brain, the cerebral cortex is the center of integral neural activity, and plays a critical role in high-order functions such as intention, memory, language and creativity. The neocortex comprises a large majority of the cortex and consists of 6 distinct neuronal layers (layers I–VI in the pial-ventricular direction). The embryonic cortical neuroepithelium generates the neurons specific to these layers in a sequential manner [4]–[5]. During mouse corticogenesis, the Reelin^+^ Cajal-Retzius cells in layer I are born the earliest, around embryonic day (E) 10. Cajal-Retzius cells are derived from the peripheral regions of the pallium, i.e., the hem or pallial-subpallial boundary regions, and migrate into the superficial-most zone of the neocortex [4], [6]–[8]. The neurons of layers II-VI are born sequentially in a specific manner known as an inside-out pattern [9]–[10]. During the early phase of this process, precursors of layer VI and V neurons are sequentially born and form the early cortical plate (CP) [11]–[12]. Then, during late corticogenesis, the neuronal precursors of layers IV, III and II are born and form the late CP [1], [4], [6], [8], [13]–[14]. This sequential generation of layer-specific cortical neurons is controlled by progressive commitments in cortical progenitors along the time axis [4], which is intrinsically programmed; indeed, such sequential generation of layer-specific neurons can be seen even in primary culture of isolated cortical progenitors [5].

A versatile ESC culture for forebrain differentiation is SFEBq culture (serum-free floating culture of embryoid body-like aggregates with quick reaggregation; [15]–[17]). In this culture, dissociated ESCs are reaggregated in a quantitative manner (usually 3,000 cells per aggregate for mESCs) using a 96-well culture plate that has a special surface coating to block cell-plate adhesion. The aggregates are cultured in suspension using serum-free medium that contains no or minimal growth factors. In this culture, mouse ESC cells (initially apolar) differentiate into epithelium-type polarized cells by day 3 and form a continuous epithelial sheet expressing epiblast-specific markers (e.g., Fgf5) on the surface of the aggregate [17]–[20]. In the absence of neural differentiation inhibitors such as BMP4, the epiblast-like epithelium undergoes neural conversion and starts to express early neural markers such as Sox1 during days 3–5. According to a recent study, this transition from epiblast to neuroectoderm is driven by the multi-zinc-finger transcription activator Zfp521, which works together with p300/CBP [19].

In the presence of a minimal level of growth factor signaling and in the absence of Shh signals, the neuroectoderm frequently differentiates into cortical neuroepithelium [16]. This is partly because the default direction of ESC-derived neuroectoderm is rostral forebrain, whereas the specification into caudal neural tissues requires the presence of caudalizing signals such as Wnt and retinoic acid in the culture medium [16], [21]–[22]. Probably because cortical differentiation is close to the default direction in ESC culture, several similar culture conditions have been shown to generate cortical tissues (progenitors and excitatory glutamatergic cortical neurons) from mouse and human ESCs [16], [23]–[27].

Among these cultures, SFEBq is unique in that it generates cortical neuroepithelium (NE) developing in a three-dimensional (3D) fashion. Notably, in this culture, the cortical NE forms a layered arrangement of distinct cortical neurons, which are born in a sequential manner from the progenitors [24]. However, the SFEBq culture method has a few technical problems to resolve before a faithful recapitulation of *in vivo* corticogenesis is possible. First, the differentiation efficiency into cortical NE can vary depending on an undefined component in the medium (see first paragraph of Results). Second, mESC-derived cortical NE cannot maintain its continuous epithelial structure longer than several culture days and gradually breaks into small epithelial rosettes within a week. Third, whereas the cortical NE in the rosette exhibits a stratified structure, the order of the layers does not fully follow the inside-out pattern seen *in vivo*.

In the present study, we sought to improve the first two points: differentiation efficiency and maintenance of continuous NE structure. We demonstrate that laminin-containing ECM proteins have strong promoting effects on cortical NE formation and maintenance in 3D self-organizing culture of mESCs.

## Results

### Optimized Cortical-differentiation Culture of mESCs with Laminin Treatment

In our previous study using SFEBq culture of mESCs, cortical differentiation was induced in medium containing the culture additive KSR (Knockout Serum Replacement) [15], [24]. The makeup of KSR is not rigorously defined and it contains an undefined component, lipid-rich albumin, which is albumin partially purified from fetal calf serum. One practical problem in using this medium is that the efficiency of cortical differentiation varies depending on the lot of KSR used. From our experience, 15–20% of KSR lots give a high efficiency of cortical differentiation (e.g., ∼90% of cells become Foxg1^+^), whereas a moderate efficiency (30–50%) is typically seen with other lots.

We previously showed that Wnt inhibition during the early phase of SFEBq culture increases cortical differentiation [16]. Consistent with this, treatment with the Wnt inhibitor IWP2 (10 µM, days 0–5) improved the efficiency of cortical differentiation in culture using KSR lots with a moderate efficiency (Fig. S1A shows an example of improvement from 30% to 60%; white columns).

To establish more stable culture conditions, we combined IWP2 treatment (2.5 µM; days 0–5) with a chemically defined medium (gfCDM; see Materials and Methods). Under these defined conditions, the efficiency of cortical differentiation was substantially improved (∼80%; Fig. S1A, black columns), and Foxg1::venus^+^ cells formed a single-peaked population with intense fluorescent signals in FACS analysis on day 10 (Fig. S1B; the control shows background fluorescence levels in undifferentiated *Foxg1::venus* ESC). In the following experiments, we performed SFEBq culture by culturing ESC aggregates in gfCDM-based medium supplemented with IWP2.

### Formation of Continuous NE Promoted by Laminin Treatment

**Figure 1:**
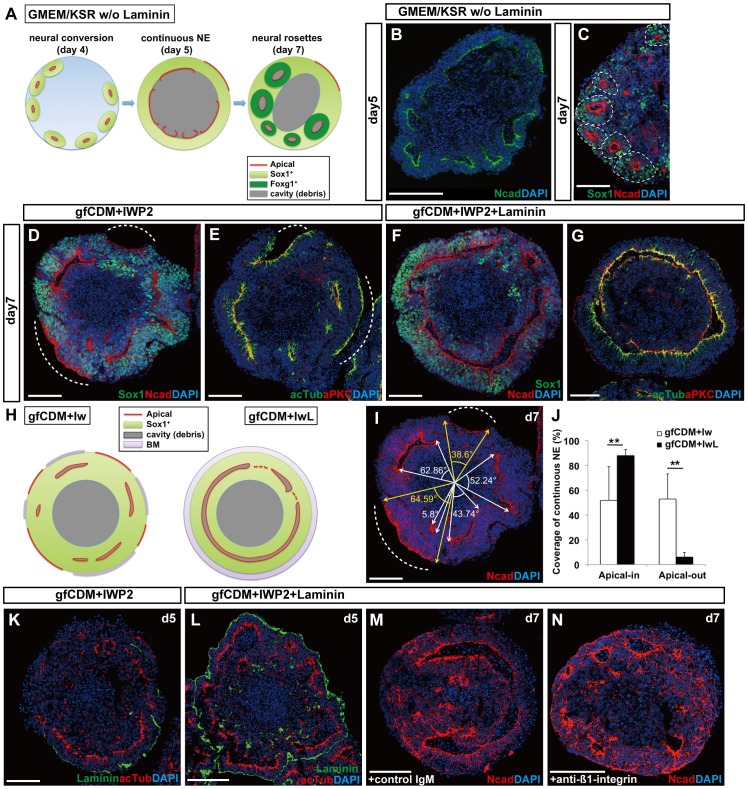
Efficient formation of continuous NE under chemically defined conditions. (A) Schematic of the formation of neural rosettes under GMEM/KSR conditions. (B) Day-5 aggregate with partially continuous NE in culture with GMEM/KSR. (C) Day-7 aggregate with neural rosettes in culture with GMEM/KSR. Immunostaining for Sox1 and N-cadherin. A dashed circle indicates the unit of a neural rosette. (D–G) Effects of laminin ECM on the consistency of the A–B polarity in NE. Immunostaining of day-7 aggregates cultured in gfCDM+IWP2 (D–E) or gfCDM+IWP2+laminin ECM (F–G) for Sox1 and N-cadherin (D,F) or acetyl-α-tubulin and aPKC (E,G). (H) Schematic of SFEBq/gfCDM+Iw and SFEBq/gfCDM+IwL culture. (I–J) Quantification of consistency of A–B polarity in NE. (I) Schematic of quantification as a sum of angular degrees. The white or yellow arrows show the ends of each apical surface of apical-in or apical-out NE, respectively. A dashed line indicates an example of apical-out NE (D–E,I). (J) The percentage of continuous NE in gfCDM+IWP2 (white bars) and gfCDM+IWP2+laminin ECM (black bars) conditions. The values shown on graphs represent the mean ± s.e.m. **P<0.01. (K–L) The effect of laminin ECM on the formation/maintenance of laminin^+^ basement membrane (day 5). (K) The continuity of laminin^+^ basement membrane was lost in day-5 aggregate cultured without exogenous laminin ECM. (L) The day-5 aggregate cultured with laminin ECM was surrounded by continunous laminin^+^ basement membrane. (M–N) The effect of laminin ECM mediated by ß1-integrin. Anti-ß1-integrin impaired the formation of continuous NE in culture with laminin ECM. Note an increase of apical-out NE (N). No substantial effect was seen with control IgM (M). BM, basement membrane. Scale bars, 100 µm.

In the original SFEBq culture for cortical differentiation, Sox1^+^ neuroepithelium (NE) gradually forms on the surface of the aggregate during days 3–5, but the continuous NE structure (seen on day 5) is relatively unstable and starts to break into small epithelial vesicles (neural rosettes) by day 7 (Fig. 1A–C) [24]. When mESCs were cultured in gfCDM-based medium supplemented with IWP2, the integrity of the Sox1^+^ NE in the aggregate was partially improved and its reformation into neural rosettes was decreased on day 7. However, this NE was not continuous with regard to its apical-basal (A–B) polarity (Fig. 1D–E). Some parts of the NE had the apical surface (aPKC^+^ and showing strong N-cad accumulation) inside, while other parts had it outside.

**Figure 2:**
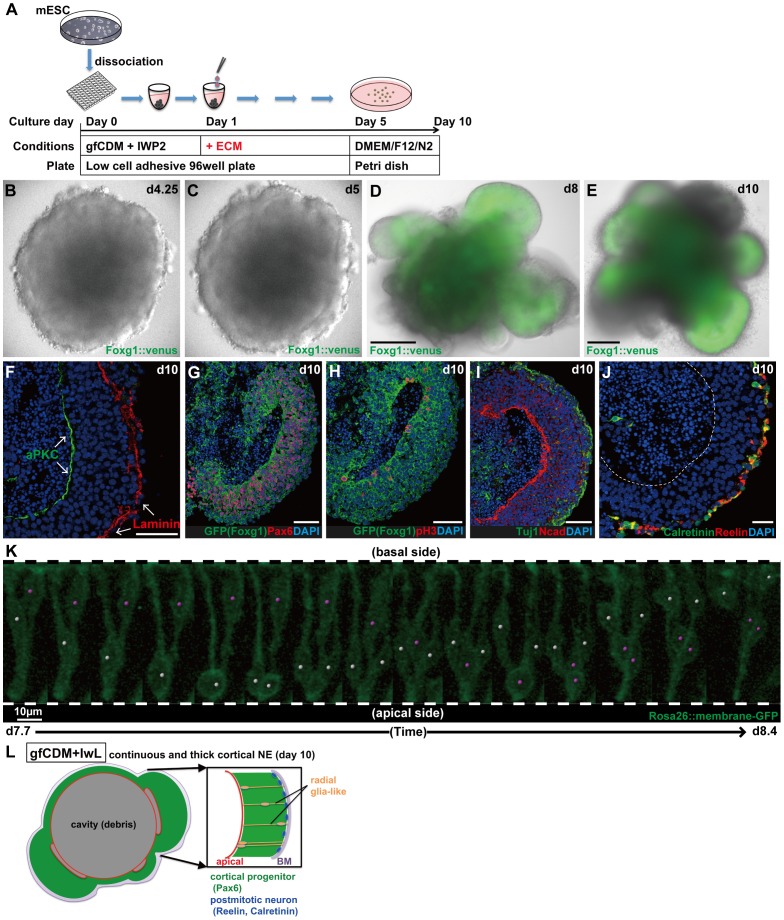
Formation of continuous cortical NE with a clear A–B polarity. (A) Schematic of SFEBq/gfCDM+IwL culture. (B–C) Live imaging showing the formation of continuous NE in SFEBq/gfCDM+IwL culture on day 4.25 (B) and on day 5 (C). (D–E) Formation of continuous NE expressing Foxg1::venus on day 8 (D) and on day 10 (E). (F–J) Immunostaining of Foxg1::venus^+^ thick NE on day 10. (F) Immunostaining for the basement membrane marker laminin and the apical marker aPKC. (G) Cortical progenitors (Foxg1::venus^+^/Pax6^+^) occupied the majority of Foxg1::venus^+^ NE. (H) Mitotic cells (pH3^+^) located on the apical surface. (I) Postmitotic neurons (Tuj1^+^) in the superficial-most layer. (J) Immunostaining for Calretinin and Reelin. (K) Live imaging for interkinetic nuclear migration in continuous cortical NE generated from mESCs. Partial labeling was done by mixing *Rosa26::membrane-GFP* ESCs with non-labeled parental EB5 cells. Snapshots of two neural progenitors (days 7.7–8.4; also shown in Movie S2) undergoing interkinetic nuclear migrations (marked with white or pink dots). (L) Schematic of the formation of continuous cortical NE under gfCDM+IwL conditions on day 10. A dashed line indicates the apical border of NE. Scale bars, 200 µm (D–E); 50 µm (F–J); 10 µm (K).

Previous studies have suggested that ECM protein components of basement membrane reinforce the stability of epithelial structures [28]. With this in mind, we next examined the effects of ECM proteins on the formation and maintenance of continuous NE. When laminin protein (200 µg/ml; BD Biosciences) was added to the culture, continuous formation of polarized NE (Sox1^+^) was reproducibly seen on day 7 in SFEBq culture using gfCDM+IWP2 medium (Fig. 1F). Importantly, the apical side of the NE in these aggregates consistently stayed inside (Fig. 1F–H). Fig. 1I shows a quantitative comparison. The large majority (>90%) of the surface NE generated with laminin had a consistent A–B polarity with the apical surface inside (Fig. 1J). In contrast, the A–B polarity of NE cultured without laminin was inverted in a substantial proportion of aggregates (∼50%). This was not due to aberrant differentiation, since the differentiation efficiency into Foxg1::venus^+^ cortical progenitors (day 10) was not largely affected regardless of the addition of laminin under the gfCDM+IWP2 conditions (Fig. S1C). Instead, the addition of exogenous laminin had a substantial impact on the integrity of basement membrane on the outer surface of the aggregate on day 5 (Fig. 1K–L). When cultured without laminin, laminin (endogenous)-positive basement membrane covered only a part of the aggregate surface (Fig. 1K, Fig. S1D–E). In contrast, the addition of laminin reinforced the integrity of basement membrane, consistent with the proper continuous arrangement of the A–B polarity (Fig. 1L, Fig. S1F–I). The continuous basement membrane was seen on day 7 and even later (shown later in Fig. 2), and contained other basement membrane components such as entactin, collagen IV and perlecan proteins (Fig S1H and data not shown). Consistent with the correlation of continuous NE formation and basement membrane coverage, treatment with the neutralizing ß1-integrin antibody, but not control IgM, impaired the formation of proper NE structure (both the integrity of the continuious NE and the consistency of the A–B polarity) in culture with laminin (Fig. 1M–N), suggesting that the biological effects of laminin were dependent on its receptor, ß1-integrin, at least to some extent.

These findings demonstrate that SFEBq culture in gfCDM with IWP2 and laminin (hereafter, referred to as SFEBq/gfCDM+IwL; Fig. 2A) is suitable for the development of proper NE structure with consistent A–B polarity. The laminin protein used here contained laminin 111 that was generated from cultured cells and co-purified with entactin (nidogen-1/2), a laminin-binding protein that forms basement membrane together with laminin. Laminin 111 may be replaced with some other laminin subtypes. For instance, addition of laminin 211 also promoted continuous formation of properly polarized NE, whereas laminin 511 had only marginal effects (Fig. S1J–L). In contrast, the addition of fibronectin (up to 100 µg/ml) to the medium did not improve the formation of continuous NE (Fig. S1M), and high concentrations of fibronectin tended to inhibit neural differentiation of mESCs in SFEBq culture (data not shown). The addition of Matrigel (also containing laminin 111 and entactin) also promoted the continuous formation of polarized NE in gfCDM+IWP2 medium (Fig. S1N–Q). Laminin 111 protein with entactin is referred to as ‘laminin ECM’ hereafter.

### Generation of Continuous Cortical NE

Live imaging also showed that neuroepithelial structures efficiently formed under the SFEBq/gfCDM+IwL conditions (Fig. 2B–C; Movie S1, part a). When the culture was continued, a few to several domains of continuous Foxg1::venus^+^ telencephalic epithelia (Foxg1::venus^+^) appeared by day 8 (Fig. 2D; Movie S1, part b). Importantly, unlike the original SFEBq method, the Foxg1::venus^+^ tissue in SFEBq/gfCDM+IwL culture did not form rosettes but maintained a continuous NE structure, even on day 10. The Foxg1::venus^+^ NE was continuous and thick, evaginating from the aggregate on day 10 (Fig. 2E). The thick NE had a clear A–B polarity with the apical surface inside, while laminin^+^ basement membrane covered the superficial surface (Fig. 2F) as seen in the embryonic cortex (Fig. S2A–B). The Foxg1::venus^+^ NE expressed Pax6 (a dorsal telencephalon marker) almost throughout the epithelial thickness, indicating that these portions were largely composed of cortical progenitors (Fig. 2G). As in the embryonic cortex, mitosis (marked by phosphorylated histone 3; pH3, Fig. S2C) was found preferentially on the luminal end (Fig. 2H), and early postmitotic neurons (TuJ1^+^) were located in a thin zone beneath the outer surface (Fig. 2I). These neurons expressed Reelin and Calretinin (Fig. 2J), suggesting that they were Cajal-Retzius cells (neurons of layer I), which are first-born neurons in the embryonic cortex.

In the continuous cortical epithelium generated by SFEBq/gfCDM+IwL, NE progenitors underwent interkinetic nuclear migration along the A–B axis, as seen in the early embryonic cortex (Fig. 2K; Movie S2). In summary, the ESC-derived NE in SFEBq/gfCDM+IwL culture forms cortical NE and retains its continuity as late as day 10 (Fig. 2L, Fig. S2D).

### Formation of Layer-specific Neurons from ESC-derived Cortical NE

**Figure 3:**
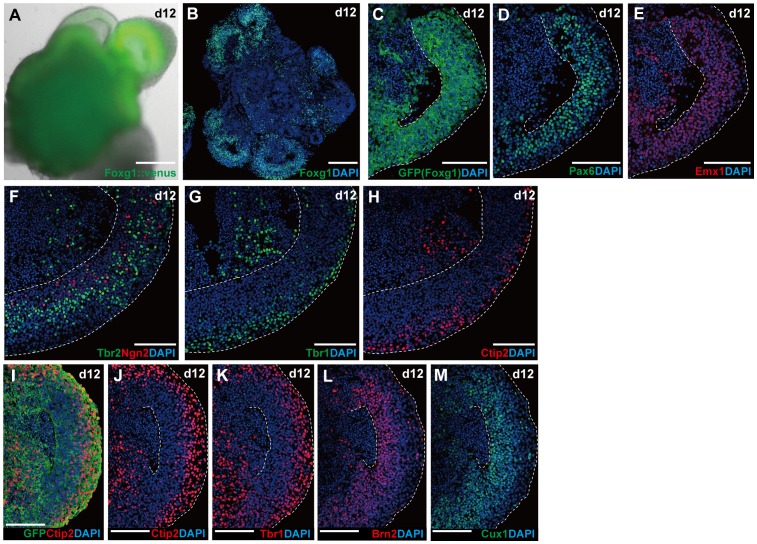
Multi-layered cortical NE on day 12. (A–B) Day-12 aggregates carrying continuous Foxg1::venus^+^ NE epithelia. (A) Fluorescent image of a Foxg1::venus^+^ aggregate. (B) Immunostaining for Foxg1. (C–H) Layered formation in continuous Foxg1::venus^+^ NE epithelia. (C–E) Cortical progenitors (Pax6^+^ and Emx1^+^) form a zone on the apical side of Foxg1::venus^+^ NE. (F) Immunostaining for Ngn2^+^ cells located in the basal part of the progenitor zone. A majority of basal progenitors (Tbr2^+^) were found outside of the Ngn2^+^ cell zone. (G–H) Early cortical plate neurons (Tbr1^+^, Ctip2^+^) were found on the basal side of Foxg1::venus^+^ NE epithelia. (I–M) Differential location of layer-specific cortical neurons in Foxg1::venus^+^ NE epithelia. (I–K) Immunostaining for Ctip2 (I–J) and Tbr1 (K). (L–M) Immunostaining for Brn2 (L) and Cux1 (M). Dashed lines indicate the apical and basal borders of NE. Scale bars, 200 µm (A–B); 100 µm (C–M).

On day 12, the continuous structure of Foxg1^+^ NE was intact (Fig. 3A–B), the cells expressed the cortical markers Pax6 and Emx1 (Fig. 3C–E), and the neurons generated in the NE exhibited a layered pattern (Fig. 3D–M). Pax6^+^ cortical progenitors were preferentially located on the apical side, forming a ventricular zone–like domain (Fig. 3D). Ngn2^+^ cells in the process of cell cycle exit were found in the basal part of this progenitor domain (Fig. 3F) [29]–[30]. In contrast, the early cortical plate (CP) neurons (layers V and VI; Tbr1^+^, Ctip2^+^; Fig. S2E–F) formed a zone on the basal side (Fig. 3G–H). The basal progenitors (Tbr2^+^; [13]) were located in the transitional domain from the Pax6^+^ progenitor zone to the Tbr1^+^/Ctip2^+^ zone (Fig. 3F), as seen in the early cortical primordium (Fig. S2E).

On day 12, Foxg1^+^ NE also formed a zone containing late CP neurons (Cux1^+^, Brn2^+^; Brn2 is also expressed in their precursors) (Fig. 3I–M). In the embryonic cortex, late CP neurons migrate basally, passing the layer of the early cortical plate neurons to form layers II–IV of the superficial zone (Fig. S2F). Such an inside-out pattern of stage-specific migration failed to form in this culture. The cells expressing Cux1 and Brn2 were mostly located in the zone apical to the early cortical plate (Fig. 3I–M).

**Figure 4:**
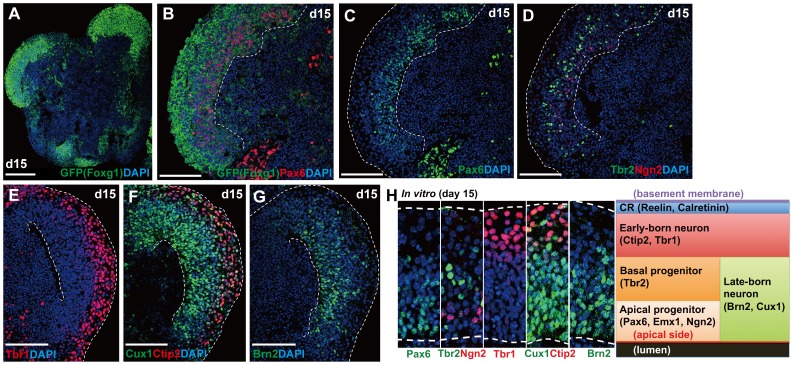
Multi-layered cortical NE on day 15. (A) Day-15 aggregate carrying continuous Foxg1::venus^+^ NE epithelia. Immunostaining for GFP. (B–G) Layer formation without the inside-out pattern. (B–C) Cortical progenitors (Foxg1::venus^+^/Pax6^+^) on the apical side. (D) Ngn2^+^ cells were located in a slightly deeper zone. Basal progenitors (Tbr2^+^) were outside of the Ngn2^+^ zone (E–G) Early cortical plate neurons (Tbr1^+^, Ctip2^+^) occupied the basal zone of continuous Foxg1::venus^+^ NE epithelia (E–F), while late cortical plate neurons (Brn2^+^, Cux1^+^) stayed on the apical side. (H) A schematic of cortical layer formation *in vitro* on day 15. Dashed lines indicate the apical or basal borders of NE. CR, Cajal-Retzius cell. Scale bars, 100 µm.

On day 15, cortical progenitors (Pax6^+^, Ngn2^+^, Emx1^+^) were still present, although they were less dense than on day 12 (Fig. 4A–D and Fig. S2G; some mitotic cells were found; Fig. S2H–M). Even at this stage, Cux1^+^ late CP neurons (also Brn2^+^ cells) stayed apical to the Tbr1^+^/Ctip2^+^ early cortical plate zone (Fig. 4E–G). Although the inside-out pattern did not form, the stratification of cortical neurons and their progenitors were clearly observed in the day-15 NE of this new culture (Fig. 4H). This stands in contrast to the previous culture, in which the A–B polarity was lost much earlier [24].

Collectively, these observations show that SFEBq/gfCDM+IwL culture promotes the formation and maintenance of continuous cortical NE, which gives rise to multi-layered structures that do not create an inside-out pattern.

### Formation and Spatial Arrangement of Non-cortical Telencephalic Tissues

**Figure 5:**
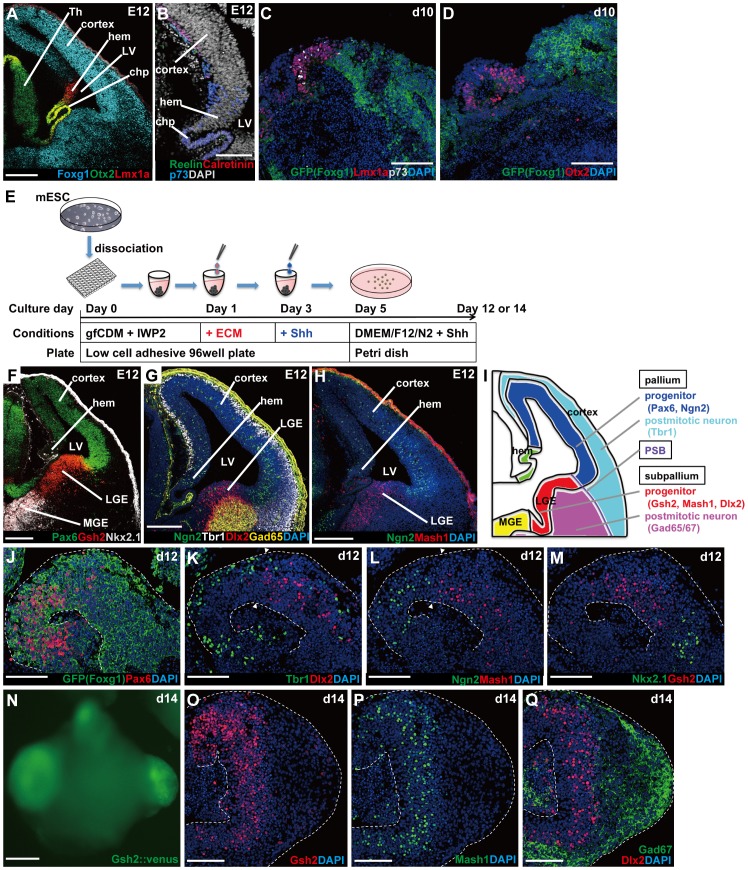
Formation and spatial arrangement of non-cortical telencephalic tissues. (A) Expression pattern of markers for cortex, cortical hem, and choroid plexus on embryonic day 12 (E12). (B) Hem-derived Cajal-Retzius cells expressing p73 on E12. (C–D) The hem-like tissues (Lmx1a^+^, Otx2^+^) formed adjacent to cortical tissues (Foxg1::venus^+^) in culture. The hem-like tissues also contained p73^+^ cells. (E) Schematic for SFEBq/gfCDM+IwL culture with Shh treatment. (F–H) *In vivo* expression of pallial and subpallial markers on E12. Cortical markers (Pax6, Ngn2, Tbr1), LGE markers (Gsh2, Dlx2, Mash1, Gad65), and MGE markers (Nkx2.1, Dlx2, Mash1, Gad65). (I) Schematic of the marker expression pattern. (J–M) Shh treatment induced the formation of subpallial tissues. LGE tissues (Gsh2^+^, Dlx2^+^, Mash1^+^) were located between cortical tissues (Pax6^+^, Ngn2^+^, Tbr1^+^) and MGE tissues (Nkx2.1^+^, Dlx2^+^, Mash1^+^). Arrowheads indicate a transition between pallial and subpallial tissues. (N) Expression of Gsh2::venus in thickened NE tissue on day 14. (O–Q) Immunostaing of Gsh2::venus^+^ NE on day 14. Immunostaining for Gsh2 (O), Mash1 (P) and Gad67/Dlx2 (Q). chp, choroid plexus; LGE, lateral ganglionic eminence; LV, lateral ventricle; MGE, medial ganglionic eminence; PSB, pallial-subpallial boundary; Th, thalamus. Scale bars, 100 µm (A–D,J–M,O–Q); 200 µm (F–H,N).

In addition to the stratification, it appears that the improved structural stability in this new 3D culture has some additional advantages for the formation of other portions of telencephanic tissues. In the embryo, the dorsal-medial border of the cortex is a special Foxg1^−^ domain that expresses Lmx1a and contains the cortical hem (consisting of Emx1^+^/Otx2^−^ and Emx1^+^/Otx2^+^ portions on dorsal and ventral sides, respectively), which abuts the choroid plexus (Emx1^−/^Otx2^+^) on its medial side (Fig. 5A–B, Fig. S3A)(this Lmx1a^+^/Foxg1^−^ domain containing these tissues is referred to as dorsal midline tissues, hereafter). In the day-10 aggregate, the lateral borders of Foxg1::venus^+^ NE were continuous with relatively thin Foxg1::venus^−^ tissues expressing Lmx1a, Otx2, and Emx1 (Fig. 5C–D), suggesting that cortical NE tissue in this culture was flanked by dorsal midline tissues (containing precursory tissues of both hem and choroid plexus types; Fig. S3B–D), as in the embryonic pallium (consistent with this, these tissues contained p73^+^ cells, which marks hem-born Cajal-Retzius cells; [31]–[32]). This finding indicated the possibility that this new culture may be also useful for studying the development of non-cortical pallial tissues.

Next, we asked whether the continuous NE generated in SFEBq-gfCDM/IwL culture could also generate subpallial (ventral telencephalic) tissues in addition to pallial tissues. To address this question, we sought to ventralize the telencephalic NE with a moderate level of Shh signals (10 nM during days 3–5 and 30 nM during days 5–14; Fig. 5E) and analyzed the culture with pallial and subpallial markers (Fig. 5F–I, Fig. S3E). Under these culture conditions, the Foxg1::venus^+^ telenephalic NE contained substantial amounts of subpallial tissues expressing lateral ganglionic eminence (LGE) markers (Mash1, Gsh2) and medial ganglionic eminence (MGE) markers (Nkx2.1; Dlx2, a marker for LGE+MGE), whereas the amount of cortical tissue (Pax6^+^, Ngn2^+^ progenitors and Tbr1^+^ neurons) reduced (Fig. 5J-5M, Fig. S3F–M). Furthermore, these subpallial tissues formed in the region adjacent to pallial tissues (Fig. 5K-5L).

To visualize the generation of LGE-like NE, we generated reporter ESC lines with *venus* cDNA knocked-in at the *Gsh2* locus (Fig. S3N). In the SFEBq culture treated with Shh, Gsh2::venus^+^ NE was seen in a few to several areas of the continuous NE (Fig. 5N). On day 14, Gsh2::venus^+^ NE formed cell masses expanding outward from the main body of the aggregate. In these masses, Gsh2^+^ LGE NE appeared as a thick zone that also expressed Mash1 (Fig. 5O–P). As seen in the embryonic LGE, the zone outside of the Gsh2^+^/Mash1^+^ NE contained numerous GABAergic neurons expressing Gad67 (Fig. 5Q). No substantial Gsh2::venus expression was observed in ESC-derived NE treated with Shh during days 3–5 and the hedgehog inhibitor cyclopamine during days 5–10 (Fig. S3O).

These observations demonstrate that SFEBq/gfCDM+IwL culture is a useful culture method for analyzing the development of cortical and non-cortical telencephalic NE tissues with a stable A–B polarity.

## Discussion

### Effect of Laminin in the Formation of mESC-derived Cortical NE

In this study, we have shown that the addition of laminin ECM promotes the formation and maintenance of continuous cortical NE with proper A–B polarity. In our culture medium, the concentration of laminin was low and the medium remained liquid. Therefore, the effect we observed was not due to mechanical stiffness of the culture environment. The addition of laminin 111 protein (co-purified with entactin) facilitated the formation and maintenance of basement membrane on the surface of mESC aggregates (Fig. 1H). Since the basement membrane also contained collagen IV and perlecan (these proteins were not added exogenously), it appears that the ESC aggregate generated these components, presumably with some help from exogenous laminin ECM. Consistent with this idea, basement membrane also forms in mESC culture without the addition of laminin (Fig. 1K), although the integrity is compromised (see fragmentation on day 7; Fig. S1E).

Entactin is essential for the integration of laminin into the collagen IV meshwork of basement membrane. In our preliminary study, laminin 111 from which entactin was stripped off (commercially available from BD as ultra-pure laminin) was much less efficient in promoting the formation of continuous NE than was the entactin-containing laminin ECM. Matrigel can be also used for the formation of continuous cortical NE under gfCDM+IWP2 conditions (Fig. S1N). Matrigel is less expensive than purified laminin ECM, but it may not be fully compatible with other medium conditions. Without IWP2 or in the presence of KSR, for example, addition of Matrigel tended to decrease cortical differentiation (our preliminary observations), suggesting that some minor ingredients other than laminin could have adverse effects in some cases.

The presence of laminin ECM clearly had an effect on the continuity of the apical-in/basal-out polarity of NE in the aggregate (Fig. 1F–G). NE generated without exogenous laminin ECM frequently contains portions with inverted A–B polarity. Furthermore, treatment with the ß1-integrin antibody strongly impaired the continuous NE formation in the presence of laminin ECM (Fig. 1N). From monolayered culture studies, it has been demonstrated that the presence and location of basement membrane can instruct the direction of A–B polarity of epithelium such that the side attached to the basement membrane becomes basal [33]. It will be an intriguing question whether the integrin signal is enough for the acting basement membrane or how the lack of the integrin signal introduces the inverted polarity. Another open question is whether laminin ECM also reinforces the NE integrity by supporting neural progenitors’ growth and maintenance, given that ECM is shown to play a role in providing a niche for neural stem cells [34].

In addition, how laminin-induced ECM signals evoke the cellular response in the forming NE remains to be clarified. As discussed above, ß1-integrin function is essential for this process. However, two different ß1-integrin ligands, laminin 111 and fibronectin, exerted distinct effects on the NE formation; unlike laminin 111, fibronectin was rather inhibitory for continous NE formation (Fig. S1J,M). This finding suggests that non-ß subunits of integrin play decisive roles in the functional specificity in this culture system. Even within the laminin family, laminin 111 and laminin 511 showed quite different potency for the promotion of NE formation, although the latter also efficiently binds to integrin receptors. It is important to understand whether this reflects a qualitative or quantitative difference between laminin 111 and 511 in integrin activation.

### Critical Roles of ECM for 3D Structural Formation *in vitro*

The present study showed that 3D ESC culture containing ECM components has an advantage in recapitulating continuous NE formation seen in embryonic development. For instance, based on the stable formation of continuous cortical NE, we recorded interkinetic nuclear migrations of cortical progenitors along the A–B axis using two-photon microscope imaging (Movie S2). With a simple partial labeling, two rounds of division cycles of radial glia-like progenitors [35] were easily observed in whole-mount imaging. Thus, this system may provide a versatile tool for many aspects of *in vitro* analysis of multicellular dynamics in corticogenesis.

In general, 3D cell culture may be performed by two approaches: free floating culture (shown in this study) and scaffold-dependent cellular engineering. Free floating culture has an advantage of larger freedom in emergence of self-driven tissue formation, as demonstrated by self-organizing eye cup formation from ESCs [36]. Another example is free floating cyst culture of MDCK cells [33], in which the epithelial sheet autonomously deform to form tubular protrusions upon HGF application [37]. Scaffold-dependent cellular engineering is exemplified by *in vitro* 3D formation of liver tissue [38] and blood vessels [39] in scaffold gel. In addition, previous studies demonstrated that epithelial-mesenchymal interactions, critical for tooth [40] and skin [41]–[42] development, are successfully recapitulated using conjugation culture in scaffold collagen gel. Importantly, in 3D skin reconstitution study, the culture contained substantial amounts of ECM proteins (e.g., collagen IV, laminin, heparan sulfate, fibronectin) produced from both keratinocytes and mesenchyme (fibroblasts), and formed well-developed basement membranes from endogenously produced ECM components [43].

In our ESC culture for cortical NE formation, the addition of laminin 111 also promoted the formation of basement membrane made up of endogenously produced ECM components (presumably by NE itself). In this viewpoint, the difference between free floating and scaffold-dependent cultures may not be fundamental but reflect the level of dependency on the internal self-organizing programs.

In the developing embryonic cortex, the basement membrane of cortical NE is continuously observed on the outer (pial) surface throughout corticogenesis. The formation and maintenance of the pial basement membrane *in vivo* depends also on meningeal mesenchymal cells, which also secrete signaling molecules (cytokines and retinoic acid) and contribute to the structural arrangement and cellular differentiation in the cortical NE [44]. It is intriguing to investigate regulatory roles of pial mesenchymal cells in cortical NE formation using our ESC culture in the future. In particular, its effects on the long-term maintenance of stem cell populations and laminar formation will be important points for such a study.

### Multi-layered Cortical NE Formation in mESC Culture

Laminin ECM, added during days 1–5, also promoted the integrity of the continuous cortical NE even beyond day 12, suggesting that initial reinforcement of basement membrane has a long-lasting effect. This maintenance of NE tissue integrity stands in clear contrast to our previous study, in which cortical NE broke into small neural rosettes around day 7. In addition to structural integrity, the continuous cortical NE in the present culture appears to grow and generate neurons more persistently. In previous conditions, the number of Pax6 cortical progenitors substantially declined after day 12, and were no longer seen by day 15. In the present culture, the cortical NE showed a large population of Pax6^+^ cells on day 12 (Fig. 3), and a reduced but still substantial population on day 15 (Fig. 4). This is not due to slowed development, because the continuous cortical NE generates late CP neurons (Cux1^+^) by day 12, as seen in previous conditions.

The improved culture method in this study enables the formation of multi-layered cortical structure from mESCs and its maintenance until day 15. However, the neuronal layers did not exhibit the proper inside-out pattern, suggesting that other factors are required, in addition to structural maintenance. This is an important question for future investigation. Candidate factors may include pial tissue, the blood vessel system, interneurons and spinocerebral fluid. They might affect migrating neuronal precursors directly or indirectly by modifying the microenvironment and radial glia. In addition, although Reelin^+^ Cajal-Retzius cells are present and form the superficial-most layer in the mESC-derived cortical NE, it remains to be seen whether they function normally in this culture.

Another intriguing question is how well this improved mESC culture for 3D telencephalic generation recapitulates the development of non-cortical telencephalic structures. For instance, cortical hem-like tissue (Otx2^+^, Lmx1a^+^, Emx1^+^, Foxg1^−^; also containing p73^+^ cells) was present at the rim of Foxg1::venus^+^ cortical NE. This interesting observation is in accord with the tissue topology of the cortical hem with the neocortex. Although we have not yet observed the generation of mature hem-associated structures such as hippocampus from mESCs, it is certainly an attractive research topic, given the importance of hippocampal neurons in physiology and pathologies such as dementia.

## Materials and Methods

### mES Cell Culture and Treatment with Soluble Factors

Mouse ES cells (EB5, *Foxg1::venus* (#1–15, #2-1)) were maintained as previously described [16]. Differentiation Medium with KSR was prepared as follows: Glasgow Minimum Essential Medium (GMEM; Invitrogen) supplemented with 5–10% Knockout Serum Replacement (KSR; invitrogen), 1 mM pyruvate (Sigma), 0.1 mM nonessential amino acids (invitrogen), 0.1 mM 2-mercaptoethanol (Sigma). Growth factor-free chemically defined medium (gfCDM) was prepared as follows: Iscove’s modified Dulbecco’s medium (IMDM; invitrogen)/Ham’s F12 medium (invitrogen) 1∶1, 1× Chemically-defined lipid concentrate (invitrogen), 450 µM monothioglycerol (Sigma), 5 mg/ml purified bovine serum albumin (>99% purified by crystallization; Sigma) and 15 µg/ml apo-transferrin (Sigma). For SFEBq culture in this study, ES cells were dissociated into single cells in 0.25% trypsin-EDTA (Invitrogen), and quickly reaggregated in differentiation medium (5000 cells per 100 µl/well) using 96-well low cell-adhesion plates (Sumilon Spheroid Plates, Sumitomo; Lipidure-coat U96w from Nunc can also be used). On day 5, cell aggregates were transferred to a 10-cm bacterial-grade dish with DMEM/F12 supplemented with N2 (Invitrogen). The day that ESCs were seeded to differentiate is defined as differentiation day 0. We examined Foxg1 induction and cortical neuronal differentiation in multiple lines of mESCs including EB5, EB3 and *Sox1::GFP* 46C cells (a derivative of the E14 line) and observed consistent results. ECM proteins were laminin/entactin complex (200 µg/ml, BD Biosciences), Matrigel (200 µg/ml, BD Biosciences), ultrapure laminin (100 µg/ml, BD Biosciences), fibronectin (50–100 µg/ml, BD Biosciences), human recombinant laminin (200 µg/ml, BioLamina, buffer exchanged into PBS and concentrated to 1 mg/ml using microcon (Millipore)). The concentrated solution of each ECM was added to each well on day 1. IWP2 (Stemgent) [45] was added to ESC suspension at 2.5 µM on day 0. Shh (R&D) was added at a final concentration of 10 nM (days 3–5) and at 30 nM (days 5–14). For an assay for integrin signal, 20 µg/ml of anti-ß1-integrin or control IgM (american hamster IgM, BD Pharmingen) was added on day 2.9 and day 4, 200 µg/ml of laminin/entactin complex was added on day 3.2, and day-7 aggregates were analyzed. Addition of Fgf8 to culture (200 µg/ml, days 3–5, R&D) is an option and tended to stabilize the efficiency of the formation of a thick NE.

### FACS Sorting

For FACS analysis, Foxg1:venus^+^ cells were counted with FACSAria (BD) and the data were analyzed with FACSDiva software (BD). Cells were dissociated into single cells by 0.25% trypsin-EDTA treatment and analyzed at 4°C. Cells were sorted on day 10 following dissociation with 0.25% trypsin-EDTA and filtration through Cell Strainer (BD Biosciences). Venus^+^ and Venus^−^ cells were gated by referring to scattered plots of the *Foxg1::venus* ESC population to avoid cross-contamination.

### Immunostaining and Reporter Cell Lines

All animal experiments were performed in accordance with institutional (RIKEN) guidelines. Immunohistochemistry was performed as described [16]. Primary antibodies were used as follows: anti-Foxg1 (rabbit/1∶3000), anti-Emx1 (guinea pig/1∶1000), anti-Gsh2 (guinea pig/1∶5000) [16], anti-Lmx1a (guinea pig/1∶20,000), anti-Dlx2 (guinea pig/1∶5000, kindly gifted from Kazuaki Yoshikawa, Osaka University, Osaka, Japan). Commercial antibodies were purchased from Abcam (acetyl-α tubulin (acTubulin)/mouse monoclonal/1∶1000, Ctip2/rat monoclonal/1∶30,000, Entactin/rat monoclonal/1∶500, GFP/chicken polyclonal/1∶1000), BD pharmingen (Gad65/mouse monoclonal/1∶200, Ki-67/mouse monoclonal/1∶200, Mash1/mouse monoclonal/1∶200, N-cadherin/mouse monoclonal/1∶1000, Nestin/mouse monoclonal/1∶300), Biopat (Nkx2.1 (TTF-1)/rabbit polyclonal/1∶5000), Cell signaling (Cleaved caspase-3/rabbit/1∶1200, Sox1/rabbit monoclonal/1∶200), Covance (Nestin/rabbit polyclonal/1∶500, Pax6/rabbit polyclonal/1∶2000, Tuj1/rabbit polyclonal/1∶5000), Invitrogen (ZO1/rabbit polyclonal/1∶500), Leica (Nkx2.1 (TTF-1)/mouse monoclonal/1∶5000), Millipore/Chemicon/Upstate (Gad67/mouse monoclonal/1∶1000, Laminin A chain/rat monoclonal/1∶2000, phospho-histon H3 (pH3)/rabbit polyclonal/1∶1000, Reelin/mouse monoclonal/1∶200, Tbr1/rabbit polyclonal/1∶5000, Tbr2/rabbit polyclonal/1∶500), Nacalai (GFP/rat monoclonal/1∶1000), Santa Cruz (Brn2/goat polyclonal/1∶500, Cux1/rabbit polyclonal/1∶500, Ngn2/goat polyclonal/1∶1000, p73/goat polyclonal/1∶4000, PKCζ (aPKC)/rabbit polyclonal/1∶300, Otx2/goat polyclonal/1∶200), and SWANT (Calretinin/rabbit polyclonal/1∶2000).

Generation of the *Foxg1::venus* line was described previously [24]. The *Gsh2::venus* knock-in ESC line was generated using the plasmid construct shown in Fig. S3B, in which *Venus* cDNA [46] was inserted in frame at the initial ATG of the *Gsh2* gene.

### Live Imaging and Quantification of NE Polarity

3D live imaging was performed as described [36] using specially assembled inverted microscopes (confocal or multi-photon) combined with a full-sized CO_2_/O_2_ incubator. The position of the ESC aggregate was fixed in 50% matrigel, which was then immersed in culture medium (described above) on a 3.5-cm glass-bottom dish. For confocal analysis, optical section images were obtained using a ×10 objective lens (Olympus), a spinning disk confocal system (CSU-X1, Yokogawa) and an EMCCD camera (Andor, 512×512 pixels). The incubation system is based on LCV-110 (Olympus), but the optical system including bilateral telecentric lenses was newly designed [36]; to obtain the whole aggregate view, a ×0.5 lens was interposed in the light path. For recording, ESC aggregates were embedded in 50% matrigel and cultured in differentiation medium with penicillin/streptomycin. For high-resolution multi-photon live imaging of interkinetic nuclear migration, a stack of optical section images (512×512 pixels for the X–Y plane and 2 µm for Z-axis step; typically 25 sections) was captured at each time point using a ×25 water-immersion lens (N.A. 1.05, Olympus) and multi-photon femtosecond laser (930nm; Mai-Tai DeepSee eHP, Spectra-Physics) with group velocity dispersion auto-compensation.

For quantification of the consistency of NE polarity, we examined the percentage of NE area with continuous polarity of apical-in or apical-out topology in day-7 aggregates cultured under gfCDM+Iw or gfCDM+IwL conditions. The percentage was calculated as a sum of angular degrees for apical NE from the aggregate center with each polarity (Fig. 1I).

## Supporting Information

Figure S1 (A–C) FACS analysis of Foxg1::venus^+^ cells on day 10. (A) Cultures with varying concentrations of IWP2 in GMEM/KSR (white bars) or gfCDM (black bars). Values shown on the graph represent the mean ± s.e.m (n  = 3). (B) FACS profiles of Foxg1::venus^+^ populations under the gfCDM conditions with 2.5 µM IWP2. Blue, differentiating cells on day 10; gray, negative control (undifferentiated *Foxg1::venus* ESCs analyzed in the same series of assays). (C) FACS profiles of Foxg1::venus^+^ populations on day 10. Gray, gfCDM+IWP2; blue, gfCDM+IWP2+laminin ECM. (D) Day-7 aggregates under the previous conditions did not contain substantial laminin^+^ basement membrane. (E) Day-7 aggregate in gfCDM+IWP2 conditions contained partially fragmented laminin^+^ basement membrane. (F) Effects of laminin ECM on the formation of the basement membrane on day 5. Immunostaining for Entactin (basement membrane) and ZO-1 (apical tight junction). (G–H) A–B polarity in continuous NE generated under gfCDM+IwL conditions on day 7. Immunostaining for basal markers (Laminin, Entactin) and the apical marker ZO-1. (I) A magnified view of continuous NE on day 7 showing continuous aPKC staining. (J–Q) Formation of continuous NE was also promoted by recombinant laminin 111 (200 µg/ml) (J), recombinant laminin 211 (200 µg/ml) (K), or Matrigel (200 µg/ml) (N–Q), while only marginal effects were seen with recombinant laminin 511 (200 µg/ml) (L). Continuous formation is inhibited by fibronectin (50 µg/ml) (M). Cells that failed to form NE in the aggregate underwent apoptosis (detected by active caspase-3; P–Q). Scale bars, 100 µm (D–H,J–Q); 50 µm (I).
(TIF)

Figure S2 (A–C) Marker expression in the embryonic pallium on E12. (A) Coronal section. (B–C) Magnified views of the cortical NE. Markers shown here include apical markers (N-cadherin, aPKC, acetyl-αtubulin), basement membrane markers (Laminin, Entactin), radial glial markers (Nestin), a neural lineage marker (Ngn2), a mitotic marker (pH3) and a postmitotic neural marker (Tuj1). (D) Formation of neural rosettes in culture with GMEM/KSR+laminin ECM on day 10. Immunostaining for GFP (Foxg1::venus) and Laminin. Color codes show Foxg1^+^ cells (green), Foxg1^−^ cells (blue), the apical surface (red), the basement membrane (purple), and the cavity (cell debris) (gray). (E–F) Schematic of the cortical layer formation *in vivo* at E14.5 (E) and at postnatal day (P) 1.5 (F). (G) Emx1^+^ cortical cells clustered on the apical side in thick NE of day-15 aggregate. (H–M) Mitotic cells (pH3^+^, Ki67^+^) gradually decreased in number but remained on day 15. The number of progenitor cells (Nestin^+^) behaved similarly. Immunostaing of cortical NE on day 10 (H,K), day 12 (I,L), and day 15 (J,M) in culture. CR, Cajal-Retzius cell; LV, lateral ventricle. Scale bars, 200 µm (A); 100 µm (D,G); 50 µm (B–C,H–M).
(TIF)

Figure S3 (A) Expression of cortex and dorsal midline tissue markers in the embryonic cortex on E12 (see also Fig. 5A): the cortex (Foxg1^+^/Emx1^+^/Lmx1a^−/^Otx2^−^), the hem (Foxg1^−/^Emx1^+^/Lmx1a^+^/Otx2^−^ and Foxg1^−/^Emx1^+^/Lmx1a^+^/Otx2^+^ for dorsal and ventral areas, respectively), and the choroid plexus (chp) (Foxg1^−/^Emx1^−/^Lmx1a^+^/Otx2^+^). (B–D) Lmx1a^+^ dorsal midline-like tissues contained multiple domains recognized by different sets of markers. (B) Lmx1a was expressed widely in the Foxg1^−^ tissue. (C) There was a gap (Foxg1^−/^Otx2^−^) between Otx2^+^ and Foxg1^+^ tissues. (D) Emx1 expression was observed in both Foxg1^+^ and Foxg1^−^ tissues, while the Foxg1^−^ tissue contained both Emx1^+^ and Emx1^−^ areas. (E) Expression of the pallial marker Ngn2 and the subpallial marker Mash1 in the embryonic cortex on E12. PSB, pallial-subpallial boundary. (F–I) Pax6^+^ pallial tissues occupied a large portion of ESC-derived NE under the conditions without Shh, while Gsh2 expression was minimal (arrow in I). (J–M) Shh treatment decreased Pax6 expression and increased Gsh2^+^ clusters (brackets in L). (N) Targeting vector for generating *Gsh2::venus* knock-in ESC lines. (O) No substantial Gsh2::venus expression was observed even on day 14 in culture treated with Shh during days 3–5 and cyclopamine during days 5–10. Scale bars, 200 µm (A,F–M,O); 100 µm (E); 50 µm (B–D).
(TIF)

Movie S1 Spontaneous formation of cortical NE in mESC aggregates. (part a) Bright-field DIC image of *Foxg1::venus* for days 1–5. Time interval, 30 minutes. Corresponds to Fig. 2A–B. (part b) Fluorescence image of *Foxg1::venus* overlaid with Bright-field DIC image for days 1–6. Time interval, 1 hour.
(MOV)

Movie S2 Interkinetic nuclear migration in mESC-derived cortical NE. Two-photon live imaging of two progenitors for days 7.5–8.8. Partial labeling was done by mixing *Rosa26::membrane-GFP* ESCs with non-labeled EB5 cells. Time interval, 7 minutes. Corresponds to Fig. 2K.
(MOV)
