# Supplementary figures and images for: Robust Formation and Maintenance of Continuous Stratified Cortical Neuroepithelium by Laminin-Containing Matrix in Mouse ES Cell Culture
Source: PLoS One. 2012 Dec 31;7(12):e53024. doi: 10.1371/journal.pone.0053024 (PMC3534089; doi:10.1371/journal.pone.0053024)

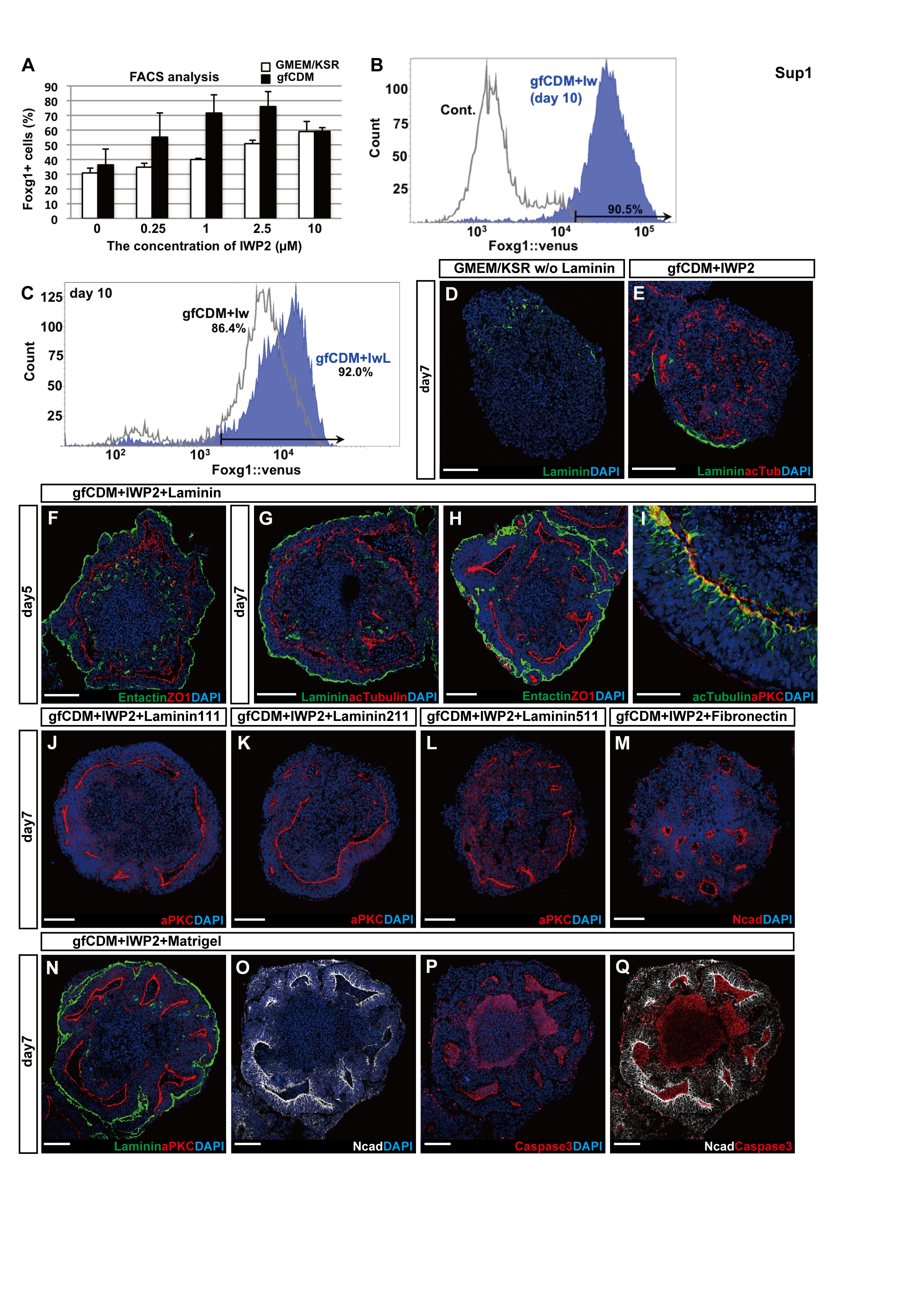

Supplement: Figure S1 — (A–C) FACS analysis of Foxg1::venus+ cells on day 10. (A) Cultures with varying concentrations of IWP2 in GMEM/KSR (white bars) or gfCDM (black bars). Values shown on the graph represent the mean ± s.e.m (n = 3). (B) FACS profiles of Foxg1::venus+ populations under the gfCDM conditions with 2.5 µM IWP2. Blue, differentiating cells on day 10; gray, negative control (undifferentiated Foxg1::venus ESCs analyzed in the same series of assays). (C) FACS profiles of Foxg1::venus+ populations on day 10. Gray, gfCDM+IWP2; blue, gfCDM+IWP2+laminin ECM. (D) Day-7 aggregates under the previous conditions did not contain substantial laminin+ basement membrane. (E) Day-7 aggregate in gfCDM+IWP2 conditions contained partially fragmented laminin+ basement membrane. (F) Effects of laminin ECM on the formation of the basement membrane on day 5. Immunostaining for Entactin (basement membrane) and ZO-1 (apical tight junction). (G–H) A–B polarity in continuous NE generated under gfCDM+IwL conditions on day 7. Immunostaining for basal markers (Laminin, Entactin) and the apical marker ZO-1. (I) A magnified view of continuous NE on day 7 showing continuous aPKC staining. (J–Q) Formation of continuous NE was also promoted by recombinant laminin 111 (200 µg/ml) (J), recombinant laminin 211 (200 µg/ml) (K), or Matrigel (200 µg/ml) (N–Q), while only marginal effects were seen with recombinant laminin 511 (200 µg/ml) (L). Continuous formation is inhibited by fibronectin (50 µg/ml) (M). Cells that failed to form NE in the aggregate underwent apoptosis (detected by active caspase-3; P–Q). Scale bars, 100 µm (D–H,J–Q); 50 µm (I). (TIF) [file pone.0053024.s001.tif]

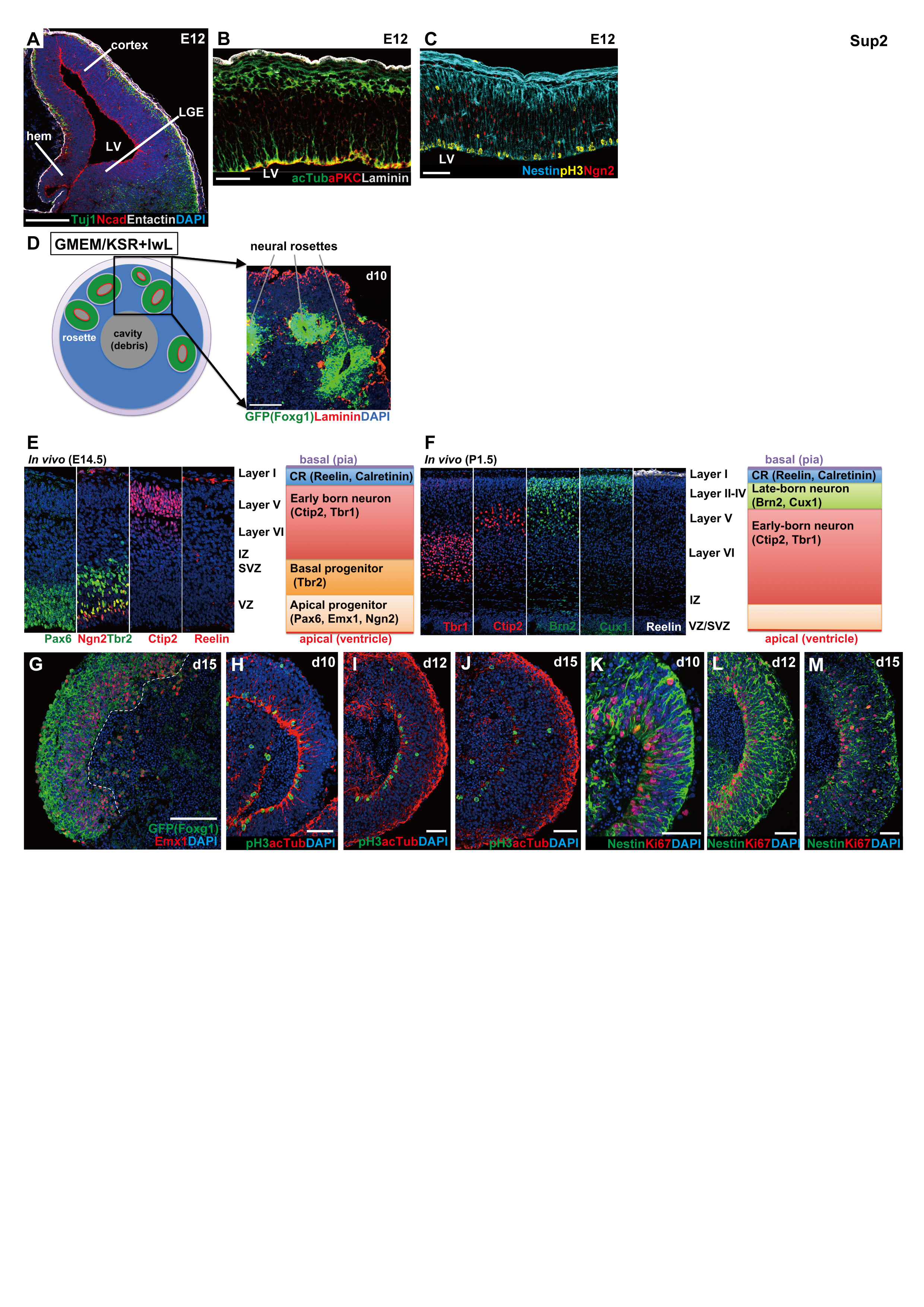

Supplement: Figure S2 — (A–C) Marker expression in the embryonic pallium on E12. (A) Coronal section. (B–C) Magnified views of the cortical NE. Markers shown here include apical markers (N-cadherin, aPKC, acetyl-αtubulin), basement membrane markers (Laminin, Entactin), radial glial markers (Nestin), a neural lineage marker (Ngn2), a mitotic marker (pH3) and a postmitotic neural marker (Tuj1). (D) Formation of neural rosettes in culture with GMEM/KSR+laminin ECM on day 10. Immunostaining for GFP (Foxg1::venus) and Laminin. Color codes show Foxg1+ cells (green), Foxg1− cells (blue), the apical surface (red), the basement membrane (purple), and the cavity (cell debris) (gray). (E–F) Schematic of the cortical layer formation in vivo at E14.5 (E) and at postnatal day (P) 1.5 (F). (G) Emx1+ cortical cells clustered on the apical side in thick NE of day-15 aggregate. (H–M) Mitotic cells (pH3+, Ki67+) gradually decreased in number but remained on day 15. The number of progenitor cells (Nestin+) behaved similarly. Immunostaing of cortical NE on day 10 (H,K), day 12 (I,L), and day 15 (J,M) in culture. CR, Cajal-Retzius cell; LV, lateral ventricle. Scale bars, 200 µm (A); 100 µm (D,G); 50 µm (B–C,H–M). (TIF) [file pone.0053024.s002.tif]

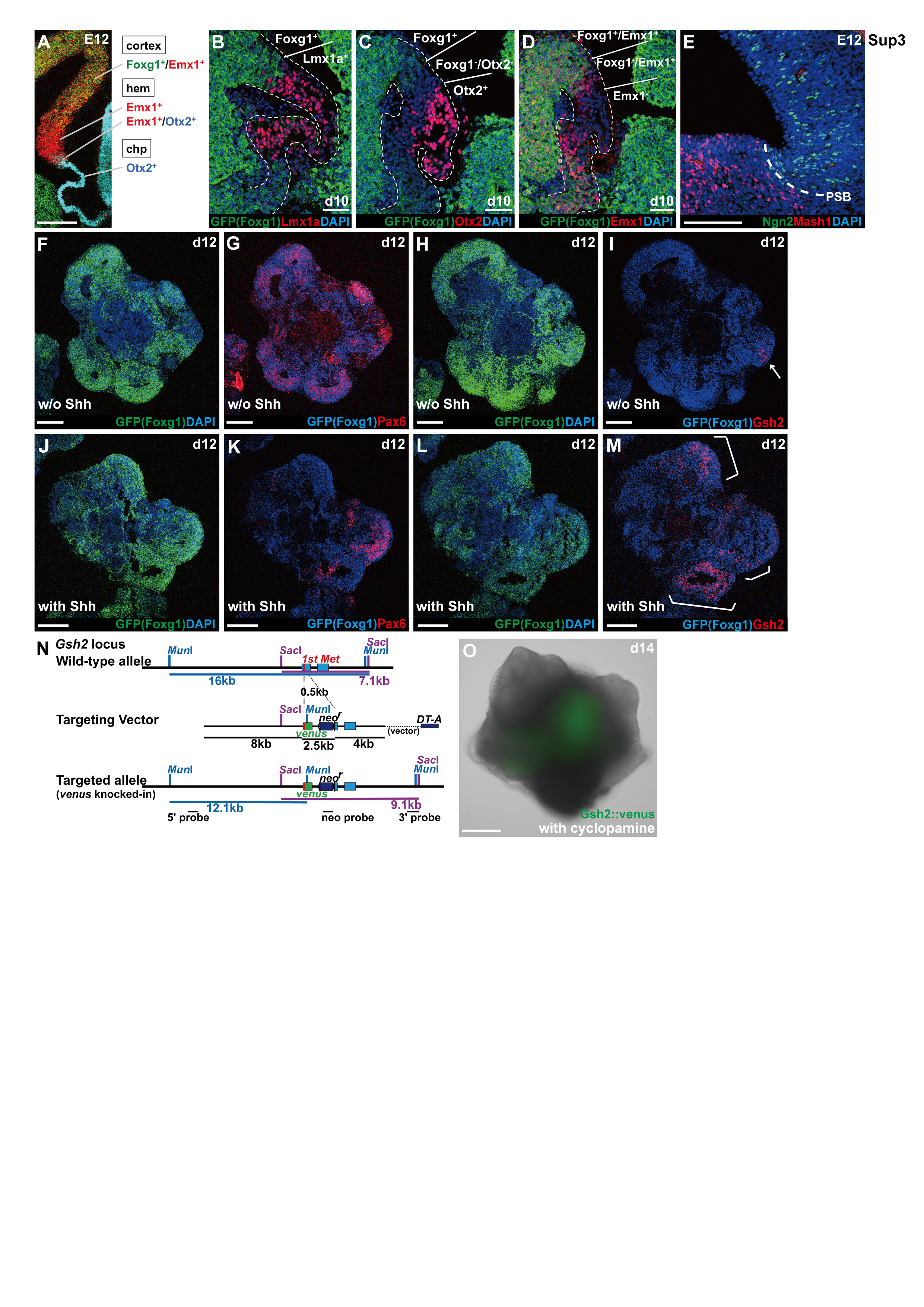

Supplement: Figure S3 — (A) Expression of cortex and dorsal midline tissue markers in the embryonic cortex on E12 (see also Fig. 5A): the cortex (Foxg1+/Emx1+/Lmx1a−/Otx2−), the hem (Foxg1−/Emx1+/Lmx1a+/Otx2− and Foxg1−/Emx1+/Lmx1a+/Otx2+ for dorsal and ventral areas, respectively), and the choroid plexus (chp) (Foxg1−/Emx1−/Lmx1a+/Otx2+). (B–D) Lmx1a+ dorsal midline-like tissues contained multiple domains recognized by different sets of markers. (B) Lmx1a was expressed widely in the Foxg1− tissue. (C) There was a gap (Foxg1−/Otx2−) between Otx2+ and Foxg1+ tissues. (D) Emx1 expression was observed in both Foxg1+ and Foxg1− tissues, while the Foxg1− tissue contained both Emx1+ and Emx1− areas. (E) Expression of the pallial marker Ngn2 and the subpallial marker Mash1 in the embryonic cortex on E12. PSB, pallial-subpallial boundary. (F–I) Pax6+ pallial tissues occupied a large portion of ESC-derived NE under the conditions without Shh, while Gsh2 expression was minimal (arrow in I). (J–M) Shh treatment decreased Pax6 expression and increased Gsh2+ clusters (brackets in L). (N) Targeting vector for generating Gsh2::venus knock-in ESC lines. (O) No substantial Gsh2::venus expression was observed even on day 14 in culture treated with Shh during days 3–5 and cyclopamine during days 5–10. Scale bars, 200 µm (A,F–M,O); 100 µm (E); 50 µm (B–D). (TIF) [file pone.0053024.s003.tif]
